# PER1 Is a Prognostic Biomarker and Correlated With Immune Infiltrates in Ovarian Cancer

**DOI:** 10.3389/fgene.2021.697471

**Published:** 2021-06-17

**Authors:** Mali Chen, Lili Zhang, Xiaolong Liu, Zhen Ma, Ling Lv

**Affiliations:** ^1^Department of Obstetrics, Gansu Province Maternity and Child-Care Hospital, Lanzhou, China; ^2^Department of Surgical Oncology, Lanzhou University Second Hospital, Lanzhou, China

**Keywords:** ovarian cancer, PER1, bioinformatics, diagnosis and prognosis biomarkers, immune infiltrates

## Abstract

**Background:** Period circadian protein homolog 1 (PER1) is an important component of the biorhythm molecular oscillation system and plays an important part in the development and progression of mammalian cancer. However, the correlations of PER1 with prognosis and tumor-infiltrating lymphocytes in ovarian cancer (OV) remain unclear.

**Methods:** The Oncomine and TIMER databases were used to examine the expression of PER1 in OV. Kaplan–Meier Plotter and PrognoScan were used to evaluate the relationship between PER1 and prognosis. Kaplan–Meier Plotter was used to analyze the relationships between PER1 and clinicopathological features of OV patients. The relationship between PER1 expression and immune infiltration in OV was investigated using the TIMER database and CIBERSORT algorithm. The STRING database was used to analyze PER1-related protein functional groups, the GeneMANIA online tool was used to analyze gene groups with similar functions to those of PER1, and Network Analyst was used to identify transcription factors that regulate PER1. The correlation between PER1 and immunoinvasion of OV was analyzed using TIMER. Finally, quantitative real-time polymerase chain reaction (qRT-PCR) was performed to detect PER1 expression.

**Results:** PER1 was differentially expressed in different cancer tissues, and its expression in various OV subtypes was lower than that in normal ovarian tissue. OV patients with low PER1 expression had a reduced overall survival rate. Decreased PER1 expression in stage 1 and stage 1+2 OV patients was related to poor prognosis, while increased PER1 expression in stage 3+4 patients and TP53 mutation were related to poor overall survival and progression-free survival. We identified eight genes whose expression was strongly correlated with that of PER1, as well as four transcription factors that regulate PER1. In OV, PER1 expression levels were positively correlated with infiltration levels of cells including neutrophils, regulatory T cells, and M2 macrophages, and closely related to a variety of immune markers. Reduced expression of PER1 was significantly associated with poor overall survival.

**Conclusion:** These findings suggest that PER1 could be used as a prognostic biomarker to determine prognosis and immune infiltration in OV patients.

## Introduction

Ovarian cancer (OV) is the fifth leading cause of cancer-related deaths in women worldwide (Dong et al., 2014). Despite advances in diagnosis, surgery, chemotherapy, and radiation therapy, OV continues to have a poor survival rate and the highest mortality rate among gynecological cancers. OV patients diagnosed at an advanced stage have poor prognosis and high recurrence rates. Determining effective markers for early diagnosis and prognosis is thus essential to reduce mortality and ensure effective therapy for OV (Bowtell et al., 2015).

Period circadian protein homolog 1 (PER1) is a core gene involved in circadian rhythm. It is located on the short arm of human chromosome 17 (17p12–p13.1) and has a total length of ~16 kb (Badiu, 2003). PER1 also has an important effect on the occurrence and development of tumors. Its main functions involve regulating the body's circadian rhythm, regulating the cell cycle, and promoting DNA damage repair. Many studies have shown that PER1 directly regulates levels of reactive oxygen species in multiple organs and regulates key effectors of energy substrate utilization in response to daily behavioral rhythms and oxidative stress, thereby maintaining the daily rhythms of mitochondrial morphology and function (Sun et al., 2020). Changes in expression levels of PER1 have been found in some cancers, including pancreatic cancer (Guo et al., 2020), oral squamous cell carcinoma (Yang et al., 2020), endometrial cancer (Wang et al., 2020), colorectal cancer (Holipah and Kuroda, 2020), and non-small-cell carcinoma (Lin et al., 2020), but this has not been studied in OV. It has also been reported that the occurrence and development of cancer, as well as its prognosis and treatment outcomes, are closely related to the abnormal expression of certain circadian clock genes (Cao et al., 2009; Sahar and Sassone-Corsi, 2009; Yang et al., 2009). Based on these findings, we propose that PER1 could be used as a prognostic indicator in OV.

We also note that circadian rhythm is closely related to the human immune system (Lange et al., 2010). Abnormalities of the biological clock contribute to exhaustion of T cells and overall upregulation of immunosuppressive molecules (including programmed death ligand 1 [PD-L1] and cytotoxic T lymphocyte-associated antigen 4 [CTLA-4]); however, the impact of PER1 on the immune environment of OV remains unknown.

In this research, we analyzed the expression levels of PER1 in OV, using the online tools TIMER and Oncomine, and studied the relationships between PER1 expression and clinicopathological parameters. The prognostic value of PER1 expression in OV was determined using Kaplan–Meier Plotter and the PrognoScan database. Quantitative real-time PCR (qRT-PCR) was used to detect PER1 expression, and patients were divided into high and low PER1 expression groups for survival analysis. In addition, we used the TIMER database to determine the correlations between PER1 and immune cell infiltration. Our findings reveal an important role of PER1 in OV and also clarify the potential relationship of PER1 with tumor immunity and its underlying mechanism.

## Materials and Methods

### Tissue Samples

We collected 60 OV cancerous and paracancerous tissue samples together with associated clinical information from Gansu Provincial Maternal and Child Health Hospital. None of the subjects underwent radiotherapy or chemotherapy prior to the surgery. All patient materials were obtained with informed consent, and this study was carried out with the approval of the Clinical Research Ethics Committees.

### qRT-PCR

TRIzol reagent (Invitrogen, Carlsbad, CA, USA) was used for total RNA extraction from tissues. RNA was reverse transcribed into cDNA using a RevertAid First Strand cDNA Synthesis Kit (Thermo-Fisher Scientific, Waltham, MA, USA). Subsequently, with the cDNA as the template, qRT-PCR was performed using SYBR Premix Ex Taq^™^ (TaKaRa, Otsu, Shiga, Japan). Relative gene expression was calculated by the 2–ΔΔCt method. GAPDH served as the internal reference. The primer sequences were as follows (“F” represents “forward”; “R” represents “reverse”). GAPDH: 5′-AGAAGGCTGGGGCTCATTTG-3′(F), 5′AGGGGCCATCCACAGTCTTC-3′(R); PER1: 5′-CTGCTACAGGCACGTTCAAG-3′(F), 5′-CTCAGGGACCAAGGCTAGTG-3′(R).

### Oncomine Database Analysis

Oncomine is currently the world's largest oncogene chip database and data mining platform, containing 715 gene expression datasets and 86,733 tumor and normal tissue samples. Here, the Oncomine database was used to detect the differential expression of PER1 between OV tissues and normal ovarian tissues. The data type was mRNA, and differential genes were identified by *t*-test with the following screening criteria: fold change >2, *P* < 0.05.

### GEPIA Database Analysis

GEPIA is a newly developed bioinformatics platform for analysis and processing of transcriptome data from The Cancer Genome Atlas (TCGA) and GTEx databases. We analyzed the correlations between PER1 and other genes using the “Correlation Analysis” module of GEPIA with *P* < 0.05 as the screening criterion.

### TIMER Database

TIMER (https://cistrome.shinyapps.io/timer/) is a database for comprehensive analysis of tumor immunity. We used the “Gene” module to analyze the correlations between PER1 gene expression and infiltration of immune cells, including CD4+ T cells, CD8+ T cells, B cells, neutrophils, macrophages, and dendritic cells. Immune cells included in the new version of TIMER showed increased infiltration levels, as presented in the results section. We used the “Diff Exp” module to detect the differential expression of the PER1 gene in different tumor tissues and normal tissues. Then, we used TIMER to detect the correlations between PER1 and immune markers of immune cells.

### Survival Analysis and Prognosis Evaluation

We used Kaplan–Meier Plotter (http://kmplot.com/analysis/) and PrognoScan (http://dna00.bio.kyutech.ac.jp/PrognoScan/index.html) for prognostic analysis. Based on the median expression of PER1, patient samples were divided into two groups for analysis with respect to overall survival (OS), disease-free survival (DFS), progression-free survival (PFS), and post-progression survival (PPS). The PrognoScan database was used to determine the prognostic value of PER1 in OV patients.

### Univariate and Multivariate Cox Regression of PER1

Univariate and multivariate Cox regression were used for survival analyses. Multivariate Cox analysis was used to compare the effects of PER1 expression and other clinical characteristics on survival. Taking the patients were divided into high and low PER1 expression groups. The statistical significance level for the two-tail test was set to 0.05.

### Protein–Protein Interaction Network Analysis

STRING (https://string-db.org/) was used to construct a PPI network, choosing the options “PER1” and “Human (Organism).”

### GeneMANIA Analysis

GeneMANIA was used to identify gene groups with similar functions to PER1 and to construct a functional network.

### Correlation Analysis of Transcription Factors and Genes

The TFs of PER1 and related genes were identified using Network Analyst (http://www.networkanalyst.ca), and their expression was analyzed using the GEPIA tool based on TCGA samples.

### Statistical Analysis

The results generated from Oncomine are displayed as *P*-values and fold change values. Kaplan–Meier Plotter, PrognoScan, and GEPIA results are presented as hazard ratios, *P*-values, and Cox *P*-values. In addition, Pearson correlation, Spearman correction, and statistical significance were used to evaluate the correlations of gene expression, and the absolute value was used to determine the strength of the correlation.

## Results

### PER1 Expression in Different Human Cancers

**Figure 1:**
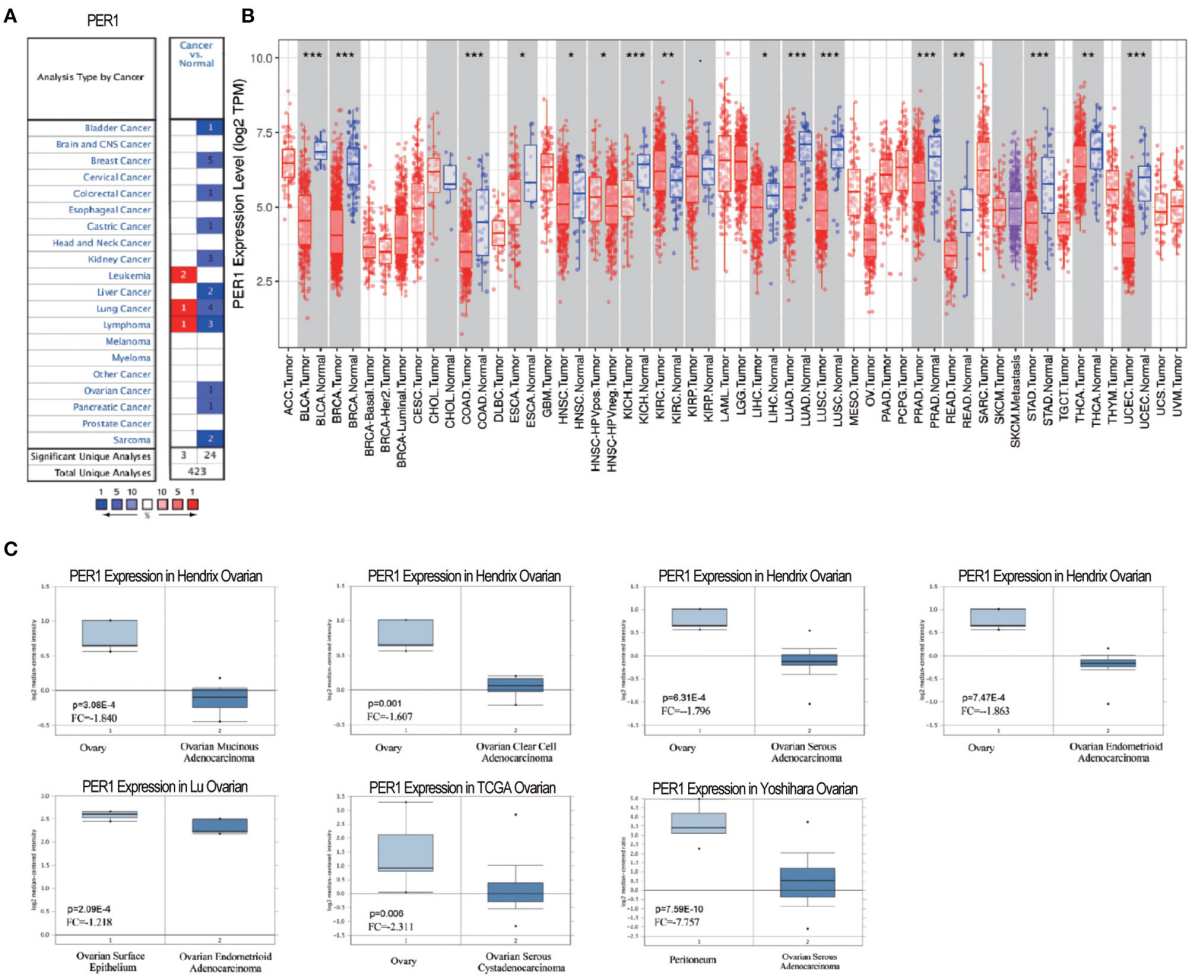
Transcription levels of PER1 in different human cancers. **(A)** Increased or decreased expression of PER1 in various cancer tissues compared with normal tissues from the Oncomine database. **(B)** PER1 expression levels in various types of cancer from the TIMER database. **(C)** Expression of PER1 is downregulated in different types of OV. Box plots comparing PER1 expression in OV patients and normal individuals constructed based on data from the Oncomine database.

We first used the Oncomine database to explore the expression levels of PER1 in different tumor types. PER1 was upregulated in three tumor types and downregulated in 24 types (Figure 1A). Then, we analyzed the expression levels of PER1 in different tumor types using the TIMER database. Compared with normal tissues, PER1 showed low expression levels in bladder cancer, breast cancer, colon cancer, kidney cancer, lung adenocarcinoma, lung squamous cell cancer, pancreatic cancer, gastric cancer, and urethral epithelial cancer tissues, while there was no control samples were available for OV (Figure 1B). We further analyzed the Oncomine data and found low expression of PER1 in mucinous ovarian adenocarcinoma, clear cell ovarian adenocarcinoma, serous ovarian adenocarcinoma, ovarian endometrioid adenocarcinoma, and ovarian endometrioid cystadenocarcinoma compared with normal ovarian tissue (Figure 1C).

### PER1 Is an Independent Prognostic Factor for OV

**Figure 2:**
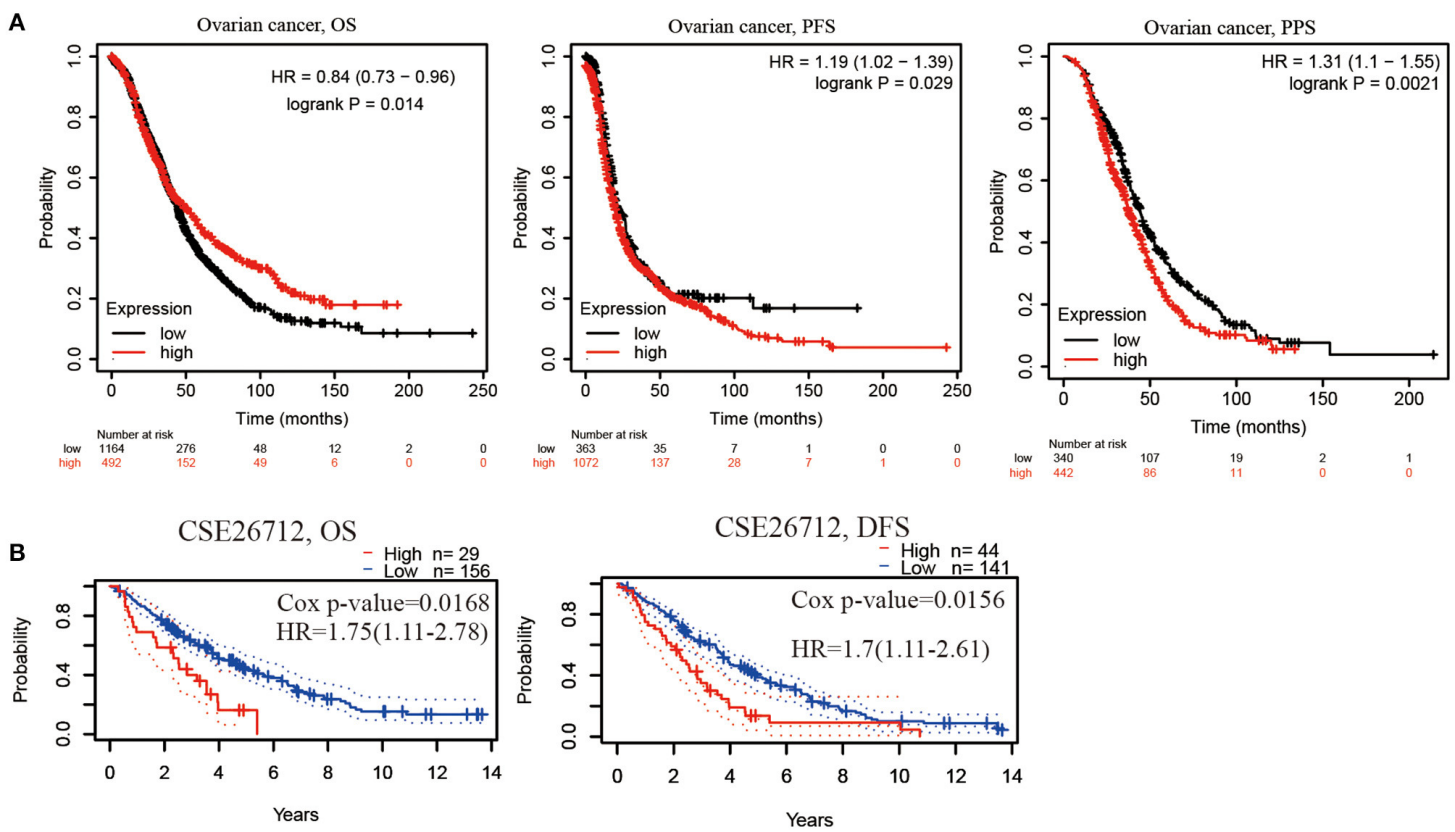
Prognostic value of PER1 mRNA expression in OV patients. **(A)** Low PER1 expression was associated with poor OS in BC patients using Kaplan–Meier Plotter, and high expression of PER1 was associated with poor PFS and PPS. **(B)** Survival curve from PrognoScan analysis for OS and DFS of patients with OV. OS, Overall survival; PFS, Progression-free survival; DFS, disease-free survival; PPS, Post Progression survival.

We analyzed the relationship between PER1 expression and the prognosis of OV patients. Kaplan–Meier analysis based on microarray data showed that low PER1 expression was associated with poor OS in OV patients (*P* = 0.014), whereas high PER1 expression was associated with poor PFS (*P* = 0.029) and PPS (*P* = 0.0021) (Figure 2A). We also assessed the prognostic potential of PER1 in OV using the PrognoScan database. In the GSE26712 cohort, high PER1 expression levels were associated with poor OS and DFS (Figure 2B). Univariate Cox analysis showed that age and PER1 were independent prognostic factors in patients with OV (Table 1); this was confirmed by subsequent multivariate Cox regression analysis (Table 2).

**Table 1:** Univariate COX regression analysis for PER1.

**Id**	**HR**	**HR.95L**	**HR.95H**	***p*-value**
Age	1.02146581	1.008809015	1.0342814	0.000841869
Grade	1.236738772	0.850976786	1.79737311	0.265299859
Stage	1.26590041	0.944222869	1.697166952	0.114962158
PER1	1.137153358	1.011959892	1.277834991	0.03079268

*HR, Hazard ratio*.

**Table 2:** Multivariate COX regression analysis for PER1.

**Id**	**HR**	**HR.95L**	**HR.95H**	***p*-value**
Age	1.022023712	1.009170894	1.035040223	0.000741433
Grade	1.143213873	0.783174087	1.668770686	0.487968691
Stage	1.262130053	0.932519954	1.708244701	0.131671624
PER1	1.126446708	1.000510744	1.268234441	0.049021419

*HR, Hazard ratio*.

### Confirmation of the Prognostic Value of PER1 in OV Based on Clinical Characteristics

**Figure 3:**
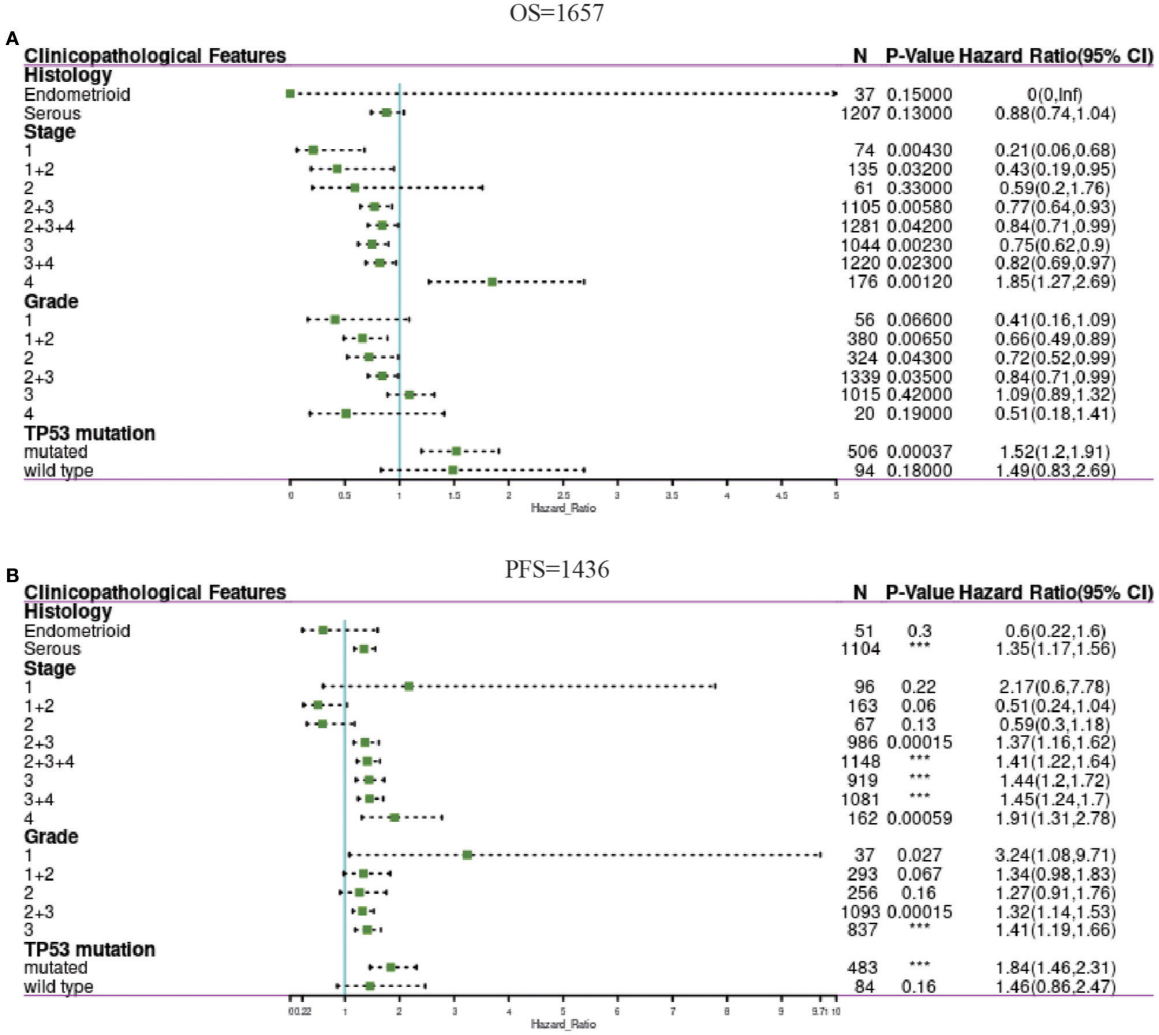
Forest plots showing associations between PER1 expression and clinicopathological features in OV patients. The above table show correlation of PER1 expression and OS in OV with different clinicopathological factors **(A)**. The below table show correlation of PER1 expression and FPS in OV with different clinicopathological factors **(B)**. ^***^P < 0.001.

To further understand the prognostic value of PER1 expression in OV, we used Kaplan–Meier analysis to determine the relationship between PER1 mRNA expression and clinical characteristics of OV patients. In the risk model for PER1 and OS, PER1 expression in OV stages 1, 1+2, 2+3, 2+3+4, 3, and 4, and grades 2, 2+3, and 3, PER1 low expression and OV patients. Poor prognosis (in terms of OS) was significantly related; in stage 3+4, TP53 mutation and high expression of PER1 were significantly related to poor prognosis (OS) in OV patients (Figure 3A).

In the risk model for PER1 and PFS, PER1 was expressed in ovarian serous tumors, stage 2+3, stage 2+3+4, stage 3, stage 4, and stage 3+4 OV, and TP53 mutant OV. High expression of PER1 was significantly correlated with poor prognosis (PFS) of OV patients (Figure 3B).

### Identification of Key Candidate Gene Networks Based on PER1 Interactions

**Figure 4:**
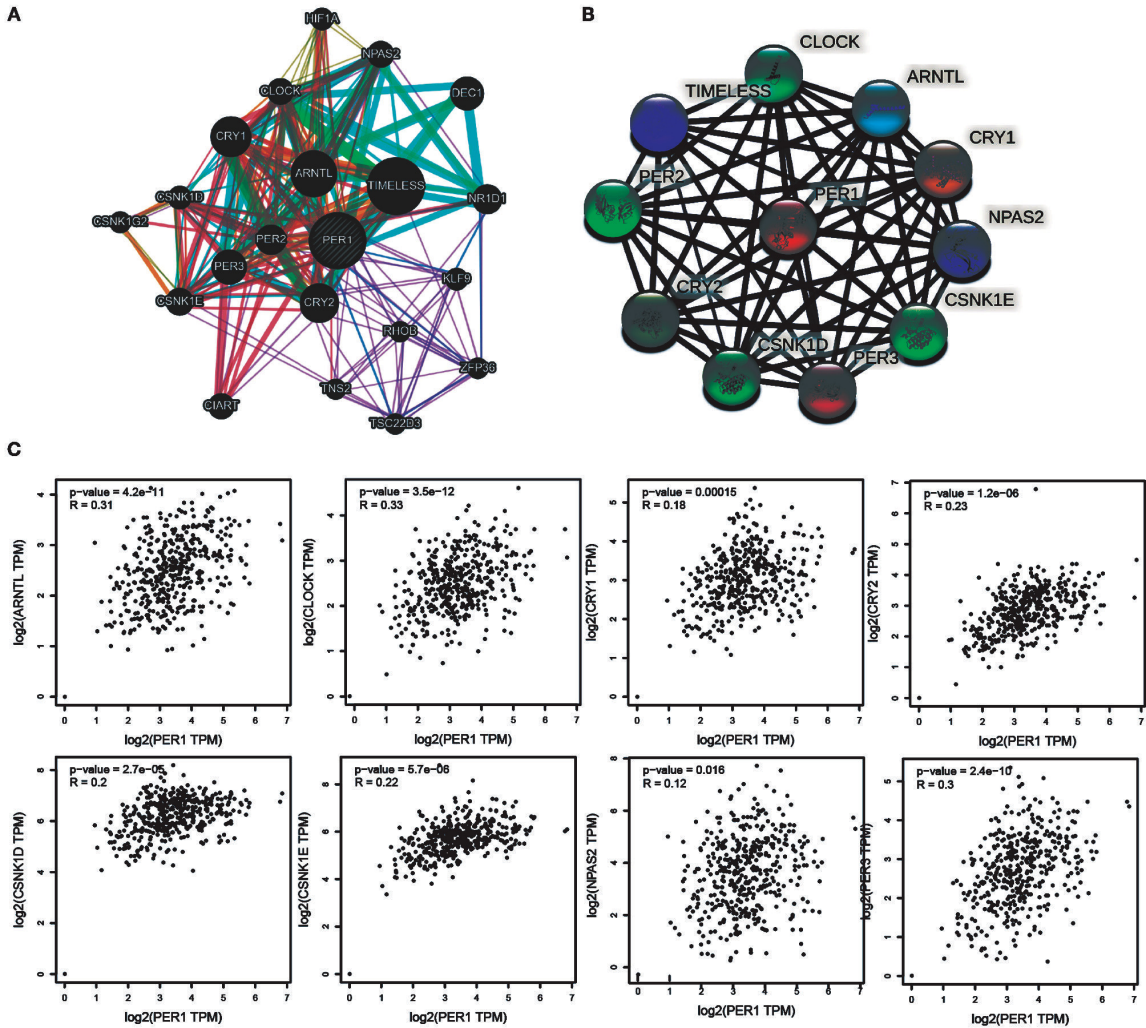
Interaction analysis for PER1 at the gene and protein levels. **(A)** Gene–gene interaction network for PER1 constructed using the GeneMANIA. The 24 most frequently changed neighboring genes are shown. Each node represents a gene. The node color represents the possible functions of the respective gene. **(B)** PPI network containing 10 nodes, constructed using the STRING database. **(C)** Scatter plots of correlations between PER1 expression and ARNTL, CLOCK, CRY1, CRY2, CSNK1D, CSNK1E, NPAS2, and PER3 in OV.

We constructed a gene–gene interaction network for PER1 to investigate its mechanism in OV and analyzed the function of these genes using the GeneMANIA database. The network contained 24 nodes representing genes that were highly correlated with PER1 (Figure 4A). In order to further explore the function of PER1, the STRING database was used to construct a PPI network. A total of 10 proteins with potential interactions with PER1 were filtered and screened, forming a very complex network (Figure 4B). We also found that the PER1 interaction network contained 10 common PER1-interacting genes across the GeneMANIA and STRING databases: CLOCK, TIMELESS, PER2, CRY2, CSNK1D, PER3, CSNK1E, NPAS2, CRY1, and ARNTL. We further evaluated the correlations between PER1 and these 10 interacting proteins based on the GEPIA database and found that the expression of PER1 in ovarian cancer was correlated with that of ARNTL, CLOCK, CRY1, CRY2, CSNK1D, CSNK1E, NPAS2, and PER3 (Figure 4C).

### Analysis of TFs and PER1-Related Genes

**Figure 5:**
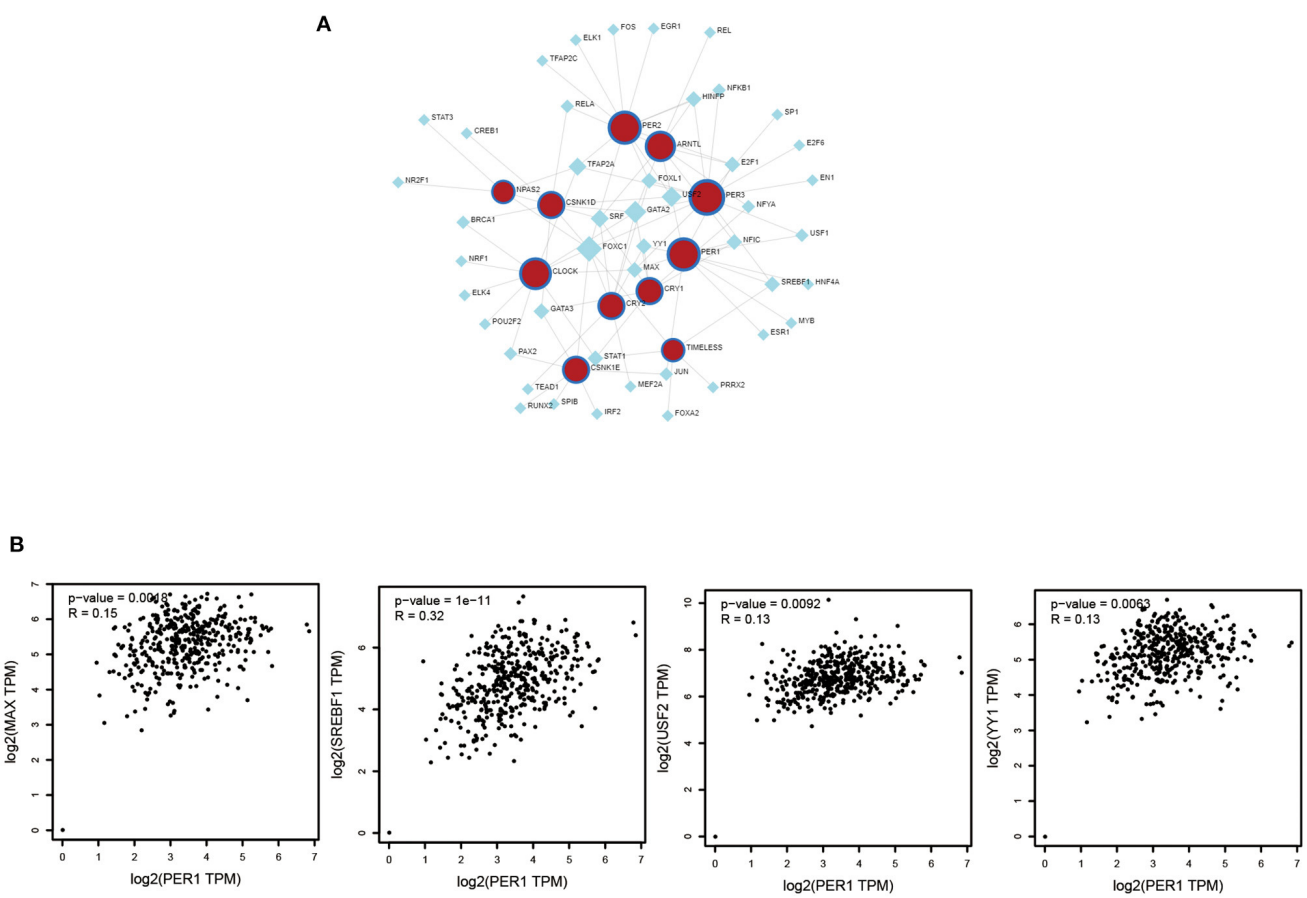
Associations of TFs and PER1-interacting genes (ARNTL, CLOCK, CRY1, CRY2, CSNK1D, CSNK1E, NPAS2, and PER3). **(A)** Network of TFs and selected PER1-interacting genes. **(B)** Correlation between TFs and PER1 expression.

To further study PER1, we explored the molecules that could regulate it and its related genes. We used the Network Analyst tool to predict TFs that could regulate PER1, ARNTL, CLOCK, CRY1, CRY2, CSNK1D, CSNK1E, NPAS2, and PER3, and constructed a PER1–TF network (Figure 5A). We found that four TFs (MAX, SREBF1, USF2, and YY1) could regulate the expression of PER1 and their expression was strongly correlated with that of PER1 (Figure 5B).

### Correlations Between PER1 Expression and Immune Cell Infiltration in OV

**Figure 6:**
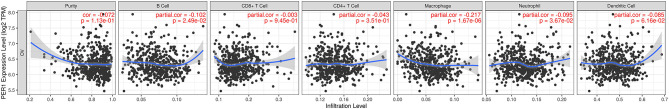
Correlation of PER1 expression with immune infiltration levels in OV. PER1 expression was significantly negatively related to infiltrating levels of macrophages, neutrophils, and B cells.

Tumor-infiltrating immune cells are independent predictors of cancer prognosis. Therefore, it was of great significance to analyze the relationships between the expression of PER1 and the infiltration levels of immune cells. As shown in Figure 6, using the TIMER database, we found that PER1 expression was significantly negatively correlated with the infiltration of B cells, macrophages, and neutrophils. In order to further explore the effects of PER1 expression on immune cell infiltration in OV, we used the “Gene” module in TIMER2.0 to search the database, inputting the target gene as PER1 and selecting OV. This module displays immune infiltration results obtained by different methods, including TIMER, EPIC, MCP-COUNTER, CIBERSORT, CIBERSORT-ABS, QUANTISEQ, XCELL, QUANTISE, and TIDE.

The results are shown in Table 3, where “+” indicates that PER1 is positively correlated with specific immune cells in OV, and “-” indicates that PER1 is negatively correlated with specific immune cells in OV. The result is the correlation index Rho (italics and black indicate a statistically significant positive correlation, black and underlined indicates a statistically significant negative correlation). PER1 expression in OV was positively correlated with neutrophils, regulatory T cells (Tregs), M2 macrophages, cancer-associated fibroblasts, naïve B cells, activated dendritic cells, and resting CD4+ memory T cells, and negatively correlated with B cells and M1 macrophages. The different methods used for CD4+ cells and macrophages led to inconsistent conclusions; this needs to be further explored.

**Table 3:** PER1 expression and infiltration levels of immune cells.

**TIMER**	**EPIC**	**MCP-COUNTER**	**CIBERSORT**	**CIBERSORT-ABS**	**QUANTISEQ**	**XCELL**	**QUANTISE**	**TIDE**
(a)	(b)	(c)	(d)	(e)	(f)	(g)	(h)	(i)
Cancer associated fibroblast	0.185(b)	0.074(g)	0.189(i)	0.18(c)				
Neutrophil	0.236(a)	0.405(c)	0.107(h)	0.04(g)	−0.054(d)	−0.031(e)		
Treg	0.25(f)	0.196(g)	−0.018(d)	0.059(e)				
Macrophage M2	0.148(h)	−0.029(g)	−0.093	−0.076(d)	0.091(e)			
B cell naïve	0.168(e)	0.044(g)	0.119(d)					
Dentritic cell activated	0.129(e)	−0.015(g)	0.118(d)					
T Cell CD4+ memory resting	0.208(e)	0.098(d)						
Macrophage	0.219(a)	−0.016(b)	–0.139(g)					
T Cell CD4+	0.298(b)	0.029(a)	0.065(d)	–0.143(h)				
Monocyte	0.128(c)	0.012(g)	0.014(d)	0.075(e)	–0.16(f)			
Macrophage M1	0.241(h)	−0.049(e)	–0.145(g)	–0.147(d)				
B Cell	0.188(h)	0.094(b)	0.002(g)	0.105(c)	−0.11(d)	–0.157(a)		
T Cell CD8+	0.116(a)	−0.033(b)	−0.065(c)	−0.108(d)	0.003(e)	−0.071(f)	−0.047(g)	
Dentritic cell	−0.011(a)	−0.031(g)	0.082(c)	0.032(f)				
T Cell CD4+ memory activated	−0.041(d)	−0.04(e)						
T Cell folicular helper	−0.002(d)	0.097(e)						

*PER1 expression in OV was positively correlated with infiltration of neutrophils and Tregs, and negatively correlated with that of B cells and M1 macrophages*.

### Correlation Analysis Between PER1 and Immune Cell Markers

In order to further explore the relationship between PER1 and the infiltrating immune cell subsets in OV, we used the TIMER database again to explore the correlations between PER1 and different immune cell markers. As shown in Table 4, the expression of PER1 was significantly correlated with various immune cell markers. In addition, we studied the correlation between PER1 and different T cell subgroups of maker (Table 5). The subgroups included Th1, Th1-like, and Th2 cells; Tregs, resting Tregs, and effector Tregs; and effector, naive, effector memory, resistant memory, and exhausted T cells. These results indicate that PER1 is closely related to immune cell infiltration in OV and suggest that it has an important role in the tumor microenvironment of OV.

**Table 4:** Correlation analysis between PER1 and immune cell markers.

**Description**	**Gene markers**	**OV**			
		**None**		**Purity**	
		**Cor**	***P***	**Cor**	***P***
CD8+ T cell	CD8A	0.082	0.155	−0.006	0.924
	CD8B	0.011	0.843	−0.065	0.307
T cell (general)	CD3D	0.015	0.795	−0.095	0.136
	CD3E	0.1	0.0816	0.003	0.966
	CD2	0.067	0.244	−0.029	0.644
B cell	CD19	0.102	0.0777	0.139	^*^
	CD79A	0.094	0.103	0.036	0.57
Monocyte	CD86	0.13	^*^	0.044	0.491
	CSF1R	0.214	^***^	0.134	^*^
TAM	CCL2	0.054	0.35	−0.036	0.567
	CD68	0.159	^**^	0.068	0.285
	IL10	0.134	^*^	0.105	0.0975
M1	IRF5	0.128	^*^	0.075	0.239
	NOS2	0.18	^**^	0.119	0.0612
	PTGS2	0.21	^***^	0.165	^**^
M2	CD163	0.248	^***^	0.199	^**^
	VSIG4	0.128	^*^	0.054	0.4
	MS4A4A	0.147	^*^	0.07	0.269
Neutrophils	CEACAM8	0.252	^***^	0.296	^***^
	ITGAM	0.256	^***^	0.189	^**^
	CCR7	0.139	^*^	0.085	0.182
Natrual	KIR2DL1	0.103	0.0737	0.051	0.42
Killer cell	KIR2DL3	0.2	^***^	0.196	^**^
	KIR2DL4	0.021	0.718	−0.009	0.889
	KIR3DL1	0.09	0.117	−0.001	0.983
	KIR3DL2	0.073	0.203	0.003	0.965
	KIR3DL3	0.116	^*^	0.126	^*^

*TAM, tumor-associated macrophage; Cor, R value of Spearman's correlation; None, correlation without adjustment; Purity, correlation adjusted by purity*.

* *P < 0.05*;

** *P < 0.01*;

*** *P < 0.001*.

**Table 5:** Correlations between PER1 and different T cell subgroups of maker.

**Description**	**Gene markers**	**OV**			
		**None**		**Purity**	
		**Cor**	***P***	**Cor**	***P***
Th1	STAT4	0.173	^**^	0.137	^*^
	STAT1	0.096	0.0951	0.105	0.0978
	IFNG	−0.007	0.91	−0.061	0.339
	TNF	0.09	0.117	0.102	0.107
Th1-like	CXCL13	0.004	0.943	−0.044	0.493
	HAVCR2	0.124	^*^	0.038	0.548
	IFNG	−0.007	0.91	−0.061	0.339
	BHLHE40	0.252	^***^	0.227	^***^
	CD4	0.167	^**^	0.084	0.187
	IL21	−0.026	0.647	0.004	0.952
Th17	STAT3	0.275	^***^	0.28	^***^
	IL17A	0.095	0.1	0.085	0.18
Treg	FOXP3	0.152	^**^	0.126	^*^
	CCR8	0.094	0.103	0.052	0.414
	STAT5B	0.357	^***^	0.328	^***^
	TGFB1	0.331	^***^	0.255	^***^
Effector T-cell	FGFBP2	0.095	0.0978	0.067	0.291
	FCGR3A	0.119	^*^	0.032	0.619
Naïve T-cell	SELL	0.072	0.211	0.009	0.886
	TCF7	0.042	0.471	0.084	0.189
Effective memory T-cell	PDCD1	0.099	0.0869	0.06	0.343
	DUSP4	0.115	^*^	0.079	0.215
	GZMK	0.091	0.112	−0.021	0.746
	GZMA	0.042	0.47	−0.049	0.442
	IFNG	−0.007	0.91	−0.061	0.339
Resistant memory T-cell	CD69	0.195	^***^	0.183	^**^
	ITGAE	0.014	0.813	−0.027	0.67
	CXCR6	0.108	0.061	0.029	0.65
	MYADM	0.316	^***^	0.237	^***^
Exhausted T-cell	HAVCR2	0.124	^*^	0.038	0.548
	TIGIT	0.103	0.0736	0.041	0.515
	CXCL13	0.004	0.943	−0.044	0.493
	LAYN	0.067	0.246	0.077	0.229
Resting Treg T-cell	FOXP3	0.152	^**^	0.126	^*^
	IL2RA	0.197	^***^	0.172	^**^
Effective Treg T-cell	FOXP3	0.152	^**^	0.126	^*^
	CTLA4	0.135	^*^	0.072	0.26
	CCR8	0.094	0.103	0.052	0.414
	TNFRSF9	0.099	0.0851	0.065	0.309
General memory T-cell	SELL	0.072	0.211	0.009	0.886
	IL7R	0.229	^***^	0.182	^**^

*Th, T helper cell; Cor, R value of Spearman's correlation; None, correlation without adjustment; Purity, correlation adjusted by purity*.

* *P < 0.05*;

** *P < 0.01*;

*** *P < 0.001*.

### PER1 Affects the Prognosis of Patients via Effects on Immune Cells

**Figure 7:**
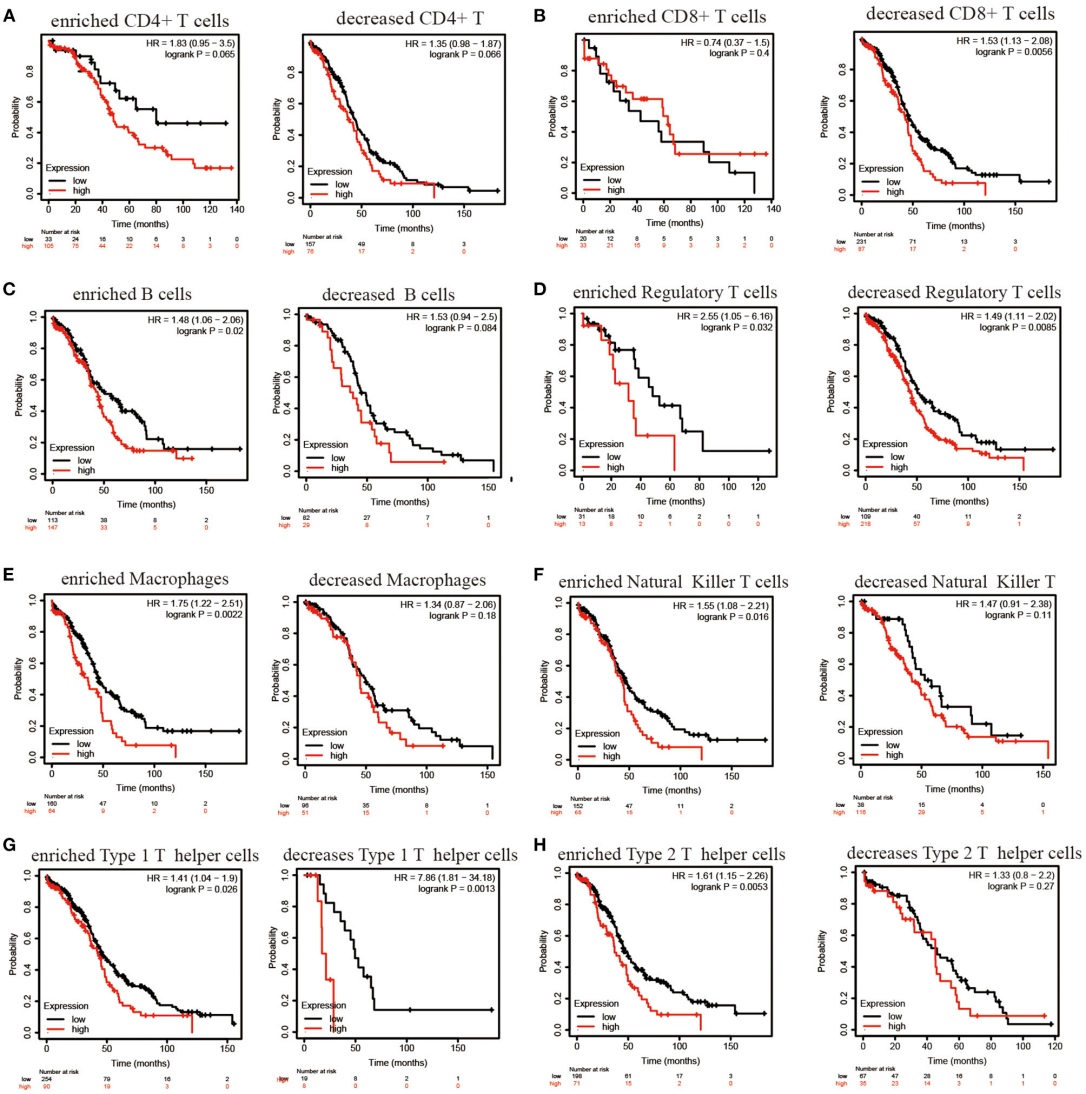
Kaplan–Meier survival curves based on expression of PER1 related to immune cell subtypes in OV. **(A–H)** Correlations between PER1 and OS in OV patients in different immune cell subgroups as estimated by the CIBERSORT algorithm.

Based on the close correlations between PER1 and immune cell infiltration, we investigated whether PER1 could affect the prognosis of patients via effects on immune cells. We stratified patients into lymphocyte-based subsets and examined the associations between PER1 and OV prognosis using Kaplan–Meier Plotter. As shown in Figure 7, decreased CD8+ memory T cells, decreased Treg cells, enriched B cells, enriched Treg cells, enriched macrophages, enriched NK T cells, enriched Th1 cells, decreased Th1 cells, decreased Th1 cells, decreased Th1 cells, decreased Th1 cells, and enriched Th2 cells, low expression of PER1 has a better prognosis. High expression of PER1 was significantly associated with poor prognosis in patients in the HTH2 cell subgroup but not in those in the CD4+ T cell subgroup (Figure 7A).

### Validation of PER1 in Independent OV Cohorts

**Figure 8:**
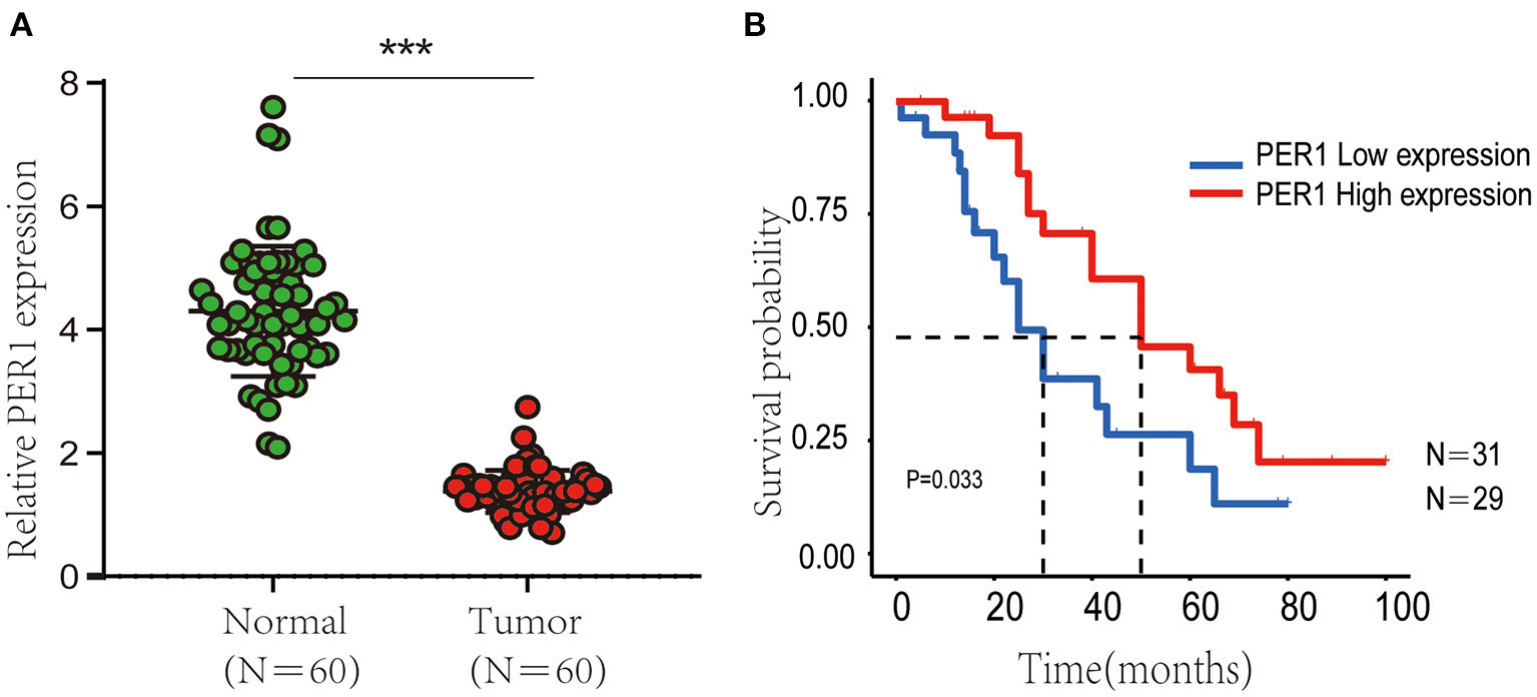
Expression of PER1 in 60 normal and tumor samples. **(A)** Kaplan–Meier OS analysis of Chinese OV patients based on expression of PER1 **(B)**. ^***^p < 0.001.

We used qRT-PCR to characterize the expression of PER1 in OV tissues and paracancerous tissues. PER1 expression in OV tissues was observably lower than that in paracancerous tissues (Figure 8A). Then, we explored the effects of PER1 on prognosis of OV patients and found that lower expression of PER1 was significantly associated with poor survival (Figure 8B).

## Discussion

One of the characteristics of malignant tumors is uncontrolled proliferation. As an important circadian rhythm gene, PER1 controls the metabolism of normal cells in the body. When it is abnormal, the resulting changes to the normal rhythm of cell proliferation lead to the occurrence of tumors. PER1 has a role in feeding and active behavior in mammals and is controlled by circadian rhythms (Kim et al., 2020). From a non-tumor perspective, PER1 is related to conditions such as Parkinson's disease, obesity, sleep, drug resistance, and premature ovarian insufficiency (Zheng et al., 2019; Delgado-Lara et al., 2020; EmeklI et al., 2020; Mabrouk et al., 2020; Arellanes-Licea et al., 2021). PER1 enhances the activity of GPX through the interaction of PER1 and GPX1 in the cytoplasm, thereby improving the oxidative phosphorylation efficiency of mitochondria (Sun et al., 2020). Current studies indicate that the clock gene PER1 is downregulated in a variety of tumors and plays an important part in promoting tumor progression. However, the biological function and mechanism of PER1 in tumors remain unknown.

Recently, studies have shown that PER1 is silenced or inhibited in a variety of cancers, and that activation or upregulation of PER1 can effectively inhibit cancer cell growth and increase spontaneous cell death. ALKBH5–PER1 is associated with the development of pancreatic cancer. Mechanistically, ALKBH5 activates PER1 in a M6a-YTHDF2-dependent manner after transcription through M6a demethylation (Liu et al., 2020). Upregulation of PER1 leads to the reactivation of ATM-CHK2-p53/CDC25c signaling, thereby inhibiting the growth of cells and the occurrence, development, and invasion of tumors. PER1 promotes the progression of oral squamous cell carcinoma by inhibiting autophagy-mediated apoptosis and enhancing cell proliferation in an Akt/mTOR-pathway-dependent manner. Low expression of PER1 is associated with poor prognosis in patients, and PER1 may be an important therapeutic target for oral squamous cell carcinoma (Yang et al., 2020). PER1 can promote cell apoptosis and expression of TNF-α, IL-6, and programmed death 1 (PD-1)/PD-L1, and inhibit tumor invasion and TUBB2B gene expression.

The severity of endometrial cancer is related to night-shift work and dysrhythmia. Rhythm-related factors PER1, TUBB2B, and tumor immune factors can regulate the pathogenesis and progression of endometrial cancer (Wang et al., 2020). As a circadian rhythm gene, PER1 has an important role in the cell cycle and affects the occurrence of cancer, including colorectal cancer (Holipah and Kuroda, 2020). In non-small-cell lung cancer, mangiferin inhibits lipopolysaccharide-induced epithelial–mesenchymal transition and enhances the expression of PER1 (Lin et al., 2020).

In this study, we investigated the expression of PER1 in OV tissues and adjacent normal tissues based on the TIMER and Oncomine databases and found that its expression in cancer tissues was lower than that in normal tissues. We used Kaplan–Meier analysis to determine the relationship between PER1 mRNA expression and the clinical characteristics of OV patients. In our models for OS and PFS, we found that PER1 was related to the stage, grade, and type of OV, and the presence of T53 mutations. Then, we evaluated the expression of PER1 and the survival of OV patients using Kaplan–Meier Plotter and PrognoScan. The results showed that PER1 was correlated with prognosis. According to the PrognoScan data, low expression of PER1 was associated with better patient prognosis in both the OS and DFS models. According to the Kaplan–Meier analysis, however, low expression of PER1 was associated with better prognosis of patients in the PPS and PFS models, whereas in the OS model high expression was associated with good prognosis. This apparent discrepancy may be related to the limited sample size; further research is needed. Overall, these results indicate that PER1 may be a valuable biomarker and an independent predictor of BC.

The development of immunotherapy strategies to eliminate cancer cells by enhancing natural defenses represents a milestone in cancer treatment. Blockade of PD-1/PD-L1 and CTLA-4 has achieved significant therapeutic success in a variety of cancers. Studies of immune infiltration have shown that the tumor immune microenvironment has a key role in cancer progression and affects the clinical outcomes of cancer patients (Murciano-Goroff et al., 2020; Zhang and Zhang, 2020). As immune cells are the cellular basis of immunotherapy, an in-depth understanding of immune infiltration in the tumor immune microenvironment can reveal underlying molecular mechanisms and provide new strategies to improve the efficacy of immunotherapy (Wu et al., 2021). A key result of the present work was that PER1 expression was closely related to the infiltration levels of a variety of immune cells. Many functional parameters in the immune system are related to cycle, including lymphocyte proliferation, natural killer cell activity, and cytokine levels (Young et al., 1995; Arjona and Sarkar, 2005; Keller et al., 2009). Inconsistent results were obtained for macrophages: PER1 expression showed different correlations with gene markers of M1 macrophages (e.g., PTGS2 and IRF5) and those of M2 macrophages (e.g., CD163, VSIG4, and MS4A4A), which may explain the potential ability of PER1 to regulate tumor-associated macrophages. In OV, there were also significant correlations between PER1 expression and the regulation of several T helper cell markers (Th1, Th2, Tfh, and Th17). These correlations indicate that PER1 could potentially regulate T cell growth in OV. Together, these findings indicate that PER1 plays an important part in the recruitment and regulation of infiltrating immune cells in OV.

This study investigated the links between PER1 and OV; however, there were some limitations. First, although we verified the expression of PER1 based on data from multiple public databases, further experiments are needed to clarify the molecular mechanism and mode of action by which PER1 regulates tumor-infiltrating cells and thereby affects the prognosis of OV patients. Second, although PER1 expression showed a certain correlation with prognosis, different results were found with different databases, possibly owing to differences in inclusion and exclusion criteria, sample size, human factors and random factors among studies; our research may be affected by the same problems and so further investigation is needed. In future experimental work, it will be necessary to address these problems. In conclusion, PER1 expression is downregulated in OV and significantly related to the pathological stage and prognosis of OV patients. PER1 and related genes have functions in the immune response. In addition, PER1 expression is related to levels of immune cell infiltration; therefore, PER1 may have a role in immunotherapy and is a potential prognostic indicator in OV.

## Data Availability Statement

The original contributions presented in the study are included in the article/supplementary material, further inquiries can be directed to the corresponding author/s.

## Ethics Statement

The studies involving human participants were reviewed and approved by Ethics Committee of Gansu Provincial Maternal and Child Health Hospital. The patients/participants provided their written informed consent to participate in this study.

## Author Contributions

MC and LZ conceived of the study and participated in design and coordination. XL drafted the manuscript. ZM collected and analyzed immune related information. LL revised manuscript. All authors read and approved the final manuscript.

## Conflict of Interest

The authors declare that the research was conducted in the absence of any commercial or financial relationships that could be construed as a potential conflict of interest.
